# The Surgical Treatment of Myoma of the Uterus

**Published:** 1899-03

**Authors:** W. Roger Williams


The Bristol

Medico-Chirurgical Journal.

MARCH, 1899.

### THE SURGICAL TREATMENT OF MYOMA OF THE UTERUS.

BY

W. ROGER WILLIAMS, F.R.C.S.

UTERINE myomata are of such common occurrence, and they so frequently give rise to serious symptoms, that every practitioner ought to be well acquainted with the chief features of the disease, and with the appropriate therapeutic indications. Modern principles of treatment differ so widely from those hitherto prevalent, that I deem no apology necessary for formulating my views on the subject.

I am not one of those who believe that the mere presence of these tumours is *ipso facto* an indication for operation; for in most cases myomata cause no dangerous symptoms, and in many others such symptoms as do occur may be relieved by palliative treatment. Uterine myomata are less dangerous to life than ovarian cystomata, and their removal is more hazardous; hence radical treatment is only exceptionally required. To justify removal there must be some urgent indication. Probably not more than one-fifth of all cases ever need active surgical intervention, but in about half of these—the patient's life being directly endangered—surgical treatment is urgently called for.

Severe and oft-repeated hemorrhage, undermining the patient's health, is an indication for operative interference, when other treatment has failed. Pressure-symptoms, indicative of dangerous complications (intestinal obstruction, peritonitis, urinary complications, etc.), whether associated with incarceration or not, may also require it. Myomata that have become inflamed, through septic infection, torsion of pedicle or otherwise, must be promptly removed. Tumours complicated with pregnancy may require surgical intervention, when they cause dystocia, and under some other circumstances. It is generally desirable to remove tumours that project into the vagina, and all very large tumours incompatible with comfortable existence. In persons near the climacteric, it is no use putting off operation, in the expectation that the tumour will subside when the catamenia cease; for this is an event that rarely happens, as is evident by noting the large proportion of patients of post-climacteric ages in any statistical list of operations.[1]

In operating for non-malignant uterine tumours, it is well to bear in mind the old surgical maxim, "*Non nocere.*" Healthy organs should not be sacrificed unnecessarily; and it is of great importance to preserve the ovaries, wholly or in part. Two contingencies must be specially provided against; viz., *sepsis* and *hemorrhage.* Strict aseptic precautions are a *sine quâ non;* for it is only by this means that successful results can be attained. For the arrest of hemorrhage, it is necessary to have accurate knowledge of the course and relations of the ovarian and uterine blood-vessels, and of the seats of election for their ligation, compression, etc.

*Removal per vaginam.*—The removal of myomata that have descended into the vagina is an ancient procedure. It was practically the only form of surgical treatment in vogue, until Amussat,[2] in 1840, opened up the cervix and removed a myoma from within the corpus uteri.

It must be borne in mind that septic infection of myomata

[1] Of 250 operated cases tabulated by Péan, 100 were between fifty and sixty years of age, and 70 between forty and fifty. (*Semaine méd.*, Avril 5, 1893.)

[2] *Mém. sur l'anat. path. des tumeurs fibreuses de l'utérus et sur la possibilité d'extirper ces tumeurs, etc.*, 1842.

projecting into the uterus and vagina is a most dangerous complication, which is apt to be caused by any form of traumatism. Consequently vaginal removal should only be attempted when complete extirpation of the tumour seems feasible. It is a mistake to attempt to imitate the natural process of expulsion by partial operation, by incision into the capsule, by decortication, by gouging or burning the tumour-substance, etc., for in nineteen out of twenty cases the natural efforts at elimination end fatally, and the results of the artificial imitation thereof are hardly less disastrous. For similar reasons, ligation and torsion of the pedicle are also to be condemned, as well as the use of the *écraseur*, the galvano-cautery snare, and the curette, for all these proceedings favour inflammation and septic infection, of which many fatal instances might be adduced. In a less degree, the same objection applies also to dilatation of the cervix by sponge tents, laminaria, etc.

The removal of myomata growing into the vagina from the inferior segment of the uterus, and of tumours that have descended from the uterus into the vagina, or that have become engaged in the cervical canal, may generally be best effected by enucleation *per vaginam*.

Pedunculated tumours of no great size are best removed by cutting through the pedicle close to the tumour with scissors curved on the flat. If feasible, the pedicle may be previously ligatured or seized with pressure-forceps. A tampon of iodoform gauze is afterwards introduced into the vagina.

As a rule, however, the class of tumours to which I am now referring do not have a distinct pedicle. Moreover, they are generally solitary, or at any rate only one has attained considerable size. They are also softer than is usual with most myomata, even when they are not of the "œdematous" variety, to which many of them belong.

Such local conditions as salpingitis, hydro-pyo- and hæmato-salpinx, pelvic and ovarian abscess, generally contra-indicate vaginal extirpation. The operation is easier in parous women than in nulliparæ. Care must be taken not to perforate the uterus, and so wound the peritoneum.

The *modus operandi* is as follows:—The field of operation having been thoroughly disinfected, and the bowels evacuated, the patient is anæsthetised and placed in lithotomy position, with Clover's crutch between the knees. The bladder is then emptied, and the vagina irrigated with antiseptic solution.[1] Simons's specula and retractors will be found invaluable for ensuring a good view of the parts.

An assistant, pressing on the hypogastric region, pushes the uterus downwards; and the surgeon, seizing the tumour with a powerful volsella, brings it down as far as possible, and with a strong blunt-pointed scissors, a crucial incision is made into its most accessible part. The severed capsule is now peeled off, a blunt enucleator being used to aid the fingers if necessary. The bared part of the tumour is next seized with the volsella, and as it is depressed the capsule is gradually separated right up to its base, when the tumour is shelled out. It may be necessary to remove large tumours piecemeal (*morcellement*), cutting them up with scissors, aided by powerful forceps, such as those introduced by Péan. Bleeding points are seized with pressure forceps, which may generally be removed before the completion of the operation. All but the stump of the capsule is cut away.

In drawing down the tumour, the uterus may be more or less inverted. Provided this is duly recognised, and care is taken to avoid wounding the displaced organ (as also the bladder, which is often displaced as well), such an occurrence facilitates the operation, and there is no difficulty in afterwards correcting the displacement. When the tumour is embedded in the substance of the inferior segment of the uterus, the overlying uterine tissues must be incised, until the capsule is exposed. The tumour is then enucleated as above described. The wound is afterwards tamponed with a long strip of iodoform gauze, which is removed a few days later.

In properly selected cases the dangers of this proceeding are slight: Leopold[2] has had a run of forty-six operations without a single fatality, and Chrobak[3] forty-three operations with but

[1] If corrosive sublimate or similar mercurial solutions be employed, they must be weak and used with caution, for many fatal cases of mercurial poisoning have resulted from the intra-uterine injection of such solutions.

[2] *Centralbl. f. Gynäk.*, 1894, xviii. 617.

[3] *Samml. klin. Vortr.*, n.F., 1892, No. 43 (*Gynäk.*, No. 17, 369.)

one death. This mode of treatment has the great advantage of removing the disease without mutilating the patient.

For the removal of tumours contained wholly within the uterine cavity, it is best to resort either to some modification of Amussat's proceeding, or to laparotomy—the intra-uterine tumour being removed by modified Cæsarean section. The former procedure is well adapted for dealing with tumours that fall short of the largest dimensions, and for inflamed and suppurating tumours ; the latter is reserved for tumours too large for removal *per vaginam*.

In vaginal extirpation, the first step is to render the tumour accessible. To effect this, the vaginal fornix is divided—anteriorly, or all round if necessary—and the bladder, having been detached from the cervix, is drawn up with a retractor, as in vaginal hysterectomy. The anterior wall of the cervix is then divided by a median longitudinal incision, and the cervical canal is laid open, until the tumour is exposed. The latter is seized with the volsella, drawn down, and deep incision is made into it. One lip of this incision is grasped with morcellation forceps, and all that it holds is cut away. By repetition of this process the whole tumour is removed piecemeal. The gravity of the operation depends much upon the size and condition of the tumour to be removed. Péan has extirpated very large tumours, such as are usually dealt with by laparotomy, in this way, with three deaths in forty operations. Vaginal hysterectomy for myomata, large enough to require removal, is an operation that is hardly ever really called for.

*Removal by Abdominal Section.*—Abdominal section ought to be regarded as a means for dealing with certain exceptional cases, rather than as the routine operative treatment. Most tumours of the largest size may be included in this category ; as well as subperitoneal tumours, especially such as are stalked and cystic ; interstitial tumours of abdominal evolution, especially when multiple and of considerable size ; intra-ligamentous myomata, and submucous intra-uterine tumours, too large for removal *per vaginam*.

A median incision is made through the abdominal wall below the umbilicus. As the bladder is often unduly elevated, its lower end should be well above the pubes; and the peritoneal sac should be first opened at its upper end. Should there be adhesions between the tumour and the great omentum, intestines, etc., these must be cautiously separated. If the union is too firm for this, omental adhesions are best overcome by excising the adherent portion of the omentum; and intestinal adhesions by excising the peritoneum covering the adherent part of the tumour. If a stalked tumour is found it may be removed, just as if it were an ovarian tumour, by transfixing the pedicle near to the uterus with a blunt needle carrying a double silk thread, each half being tied separately, and the threads cut short. The pedicle is then cut through on the distal side of the ligature, and dropped. The mortality ought not to exceed three or four per cent. As the desirability of sparing the reproductive organs whenever possible, becomes more generally recognised, the superiority of a conservative proceeding, like abdominal enucleation, over any form of hysterectomy will become apparent, especially for comparatively young women, who may bear children after this operation.

Abdominal enucleation is the best means for removing sessile subperitoneal tumours; intra-parietal tumours of abdominal evolution, especially such as grow from the fundus and anterior surface of the uterus; and intra-ligamentous myomata. When the appendages are extensively diseased, hysterectomy may be preferable.

Schröder and Martin have especially identified themselves with the improvement of this operation. The tumour, having been explored and freed from adhesions, is brought up into the wound. To prevent hemorrhage during the operation, a constricting india-rubber cord is temporarily fixed round the uterus below the tumour. To effect this, in some few cases, it may be necessary first of all to divide each broad ligament, after having ligatured the utero-ovarian blood-vessels on both sides (i.) at the pelvic margin near the infundibular pelvic ligament; (ii.) near the uterine insertion of the round ligament. The field of operation is shut off from the rest of the abdomen by packing aseptic sponges or gauze compresses—wrung out in hot sterilised water—round the uterus.

Enucleation is commenced by making a longitudinal incision into the projecting part of the tumour, so as to divide its capsule. The tumour is then seized and shelled out with the fingers, aided by a blunt enucleator. If necessary, it may be cut up and removed piecemeal. Redundant tissue about its bed is cut away, and the cavity is uniformly closed throughout by one or more rows of super-imposed buried catgut sutures. Over these the divided peritoneum is drawn, and its infolded serous margins are united by a row of Lembert's sutures. The elastic constrictor is removed, and any bleeding points are secured. The toilet of the peritoneum is performed. Lastly, the stump is examined, and when satisfactory hæmostasis has been secured, it is dropped into the abdomen, and the operation is completed as in ovariotomy.

Should the uterine cavity be opened, as frequently happens in dealing with large tumours, it may be closed with a continuous catgut suture, over which other rows of buried sutures are placed. Zweifel's[1] results show that there is no scientific basis for the prejudice against opening the uterine cavity, on account of the supposed risk of septic infection; and his experience agrees with the results of bacteriological research, which have repeatedly demonstrated the absence of pathogenic microbes from the healthy uterus. In the highly exceptional event of intractable hemorrhage taking place from the sutured pedicle, the bed of the tumour may be opened up, and plugged with a long strip of iodoform gauze. The margins of the uterine wound are then sutured to the parietal peritoneum at the lower part of the abdominal incision, on each side, the free end of the gauze tampon being brought out at its lower angle. The rest of the abdominal wound is then closed in the usual way. To make assurance doubly sure, Alexander[2] anchors the fundus uteri to the abdominal wound with silkworm-gut sutures, passed through the entire thickness of the latter, and tied externally. When the cavity of the uterus has been opened, some surgeons pack the bed of the tumour with a strip of iodoform gauze, the free end of which is passed into the vagina. Of 141 such

[1] *Centrabl. f. Gynäk.*, 1894, xviii. 321.

[2] *Brit. M. J.*, 1898, i. 1318.

operations by Martin,[1] 26 died, or 18.4 per cent., but th includes all his early operations before the *technique* was perfected; of the 20 cases reported, only 1 was lost. Engström[2] has done 100 operations, with only 5 deaths; but in more than half his cases the tumours removed were not very large. With regard to the remaining operations practised for the removal of myomata, partial hysterectomy (hystero-myomectomy) is preferable to abdominal panhysterectomy, and the latter is preferable to panhysterectomy by the "combined method"; but the truth is, the more the field of these mutilating proceedings is contracted the better.

[1] *Deutsche med. Wchnschr.*, 1893, xix. 709.

[2] *Mitth. a. d. gynaek. Klin. d. O. Engström in Helsingfors*, 1897, i. 1.

